# Immune-related lncRNAs signature and radiomics signature predict the prognosis and immune microenvironment of glioblastoma multiforme

**DOI:** 10.1186/s12967-023-04823-y

**Published:** 2024-01-26

**Authors:** Jixin Luan, Di Zhang, Bing Liu, Aocai Yang, Kuan Lv, Pianpian Hu, Hongwei Yu, Amir Shmuel, Chuanchen Zhang, Guolin Ma

**Affiliations:** 1 Department of Radiology, China-Japan Friendship Hospital, Beijing, China; 2 China-Japan Friendship Hospital (Institute of Clinical Medical Sciences), Chinese Academy of Medical Sciences & Peking Union Medical College, Beijing, China; 3 Department of Radiology, Liaocheng People’s Hospital, Shandong First Medical University & Shandong Academy of Medical Sciences, Liaocheng, Shandong China; 4 Peking University China-Japan Friendship School of Clinical Medicine, Beijing, China; 5 McConnell Brain Imaging Centre, Montreal Neurological Institute, McGill University, Montreal, QC Canada; 6 Department of Neurology and Neurosurgery, McGill University, Montreal, QC Canada

**Keywords:** Glioblastoma multiforme, LncRNAs, Radiomics, Immunity, Immune infiltration

## Abstract

**Background:**

Glioblastoma multiforme (GBM) is the most common primary malignant brain tumor in adults. This study aimed to construct immune-related long non-coding RNAs (lncRNAs) signature and radiomics signature to probe the prognosis and immune infiltration of GBM patients.

**Methods:**

We downloaded GBM RNA-seq data and clinical information from The Cancer Genome Atlas (TCGA) project database, and MRI data were obtained from The Cancer Imaging Archive (TCIA). Then, we conducted a cox regression analysis to establish the immune-related lncRNAs signature and radiomics signature. Afterward, we employed a gene set enrichment analysis (GSEA) to explore the biological processes and pathways. Besides, we used CIBERSORT to estimate the abundance of tumor-infiltrating immune cells (TIICs). Furthermore, we investigated the relationship between the immune-related lncRNAs signature, radiomics signature and immune checkpoint genes. Finally, we constructed a multifactors prognostic model and compared it with the clinical prognostic model.

**Results:**

We identified four immune-related lncRNAs and two radiomics features, which show the ability to stratify patients into high-risk and low-risk groups with significantly different survival rates. The risk score curves and Kaplan–Meier curves confirmed that the immune-related lncRNAs signature and radiomics signature were a novel independent prognostic factor in GBM patients. The GSEA suggested that the immune-related lncRNAs signature were involved in L1 cell adhesion molecular (L1CAM) interactions and the radiomics signature were involved signaling by Robo receptors. Besides, the two signatures was associated with the infiltration of immune cells. Furthermore, they were linked with the expression of critical immune genes and could predict immunotherapy’s clinical response. Finally, the area under the curve (AUC) (0.890,0.887) and C-index (0.737,0.817) of the multifactors prognostic model were greater than those of the clinical prognostic model in both the training and validation sets, indicated significantly improved discrimination.

**Conclusions:**

We identified the immune-related lncRNAs signature and tradiomics signature that can predict the outcomes, immune cell infiltration, and immunotherapy response in patients with GBM.

## Background

Glioblastoma multiforme (GBM) is the most common primary malignant brain tumor in adults, with a 5 year survival rate of 6–22%, depending on the patient’s age at diagnosis and a combination of other risk factors [1]. The prognosis of GBM may be influenced by many factors, including the patient's age, race, radiotherapy, the size, location, and histocytological composition of the tumor [2–4]. Prognostic models that only include patients’ predictors often have difficulty in accurately predicting overall patient survival. Therefore, the search for new biomarkers is crucial to improve the survival rate and reduce the burden of GBM patients.

Noncoding RNAs, including microRNAs (miRNAs) and long noncoding RNAs (lncRNAs), play a crucial role in epigenetic regulation and can serve as diagnostic markers for malignant cancers [5]. Specifically, lncRNAs are instrumental in various aspects of cancer immunity, including antigen exposure, antigen recognition, immune activation, immune cell infiltration, and immune-checkpoint blockade (ICB) [6]. Immune cell infiltration in the tumor microenvironment exhibits a large variation in GBM subtypes and patients, and these factors lead to GBM-induced immunosuppression and consequently to immunotherapy failure [7]. Therefore the identification of immune cells associated with the tumor microenvironment helps to elucidate the general mechanisms of GBM immunosuppression. In recent years, the exploration and development of cell-based immunotherapies in treating solid tumors have received considerable attentions. Immune checkpoint inhibitors (ICIs) targeting programmed cell death 1 (PD-1) or its ligand 1 (PD-L1) have achieved great clinical success in antitumor therapy [8, 9]. However, most cancer patients do not respond positively to ICB therapy [10]. Therefore, the search for effective predictive biomarkers of therapeutic response could improve the positive response rate of ICB therapy. Several studies have shown that HOTAIR [11], MALAT1 [12] and HIF1A-AS2 [13] lncRNAs are associated with the prognosis of GBM, and some researches have used related ICIs to determine the prognosis of GBM [14, 15]. Six immune-lncRNAs were combined to form a signature for GBM patients, and survival analysis revealed a significant difference between high- and low-risk groups [16]. However, the correlation between lncRNAs and immune cell infiltration and ICB in GBM is not yet known.

Radiomics, which is a technique that aims to extract the maximum amount of data from digital medical images, can assess the immune infiltration of tumors and the immune activation status of patients through data mining and analysis of medical imaging, predict the effectiveness of patients receiving immunotherapy, and as a result judge the prognosis of patients [17, 18]. Liu et al. [19]. establishes an immune cell infiltration-related prognostic biomarker and explores the associations between immune cell infiltration signatures and radiomic features in GBM patients. The results of a study showed that the CD8 + T-cell infiltration level in four different cohorts of solid tumor patients could effectively predict the efficacy of immunotherapy based on the CD8 + cell score [20]. The findings of a study on the radiomics signature's ability to predict the prognosis of gastric cancer and the immune score for the disease demonstrate that radiomics signatures can noninvasively assess the immune score for the tumor microenvironment [21]. Magnetic resonance imaging (MRI) has an important role in both diagnosis and prognosis of GBM, such as determining somatic mutations or activation of specific molecular pathways through histological features [22–24]. MR images such as contrast-enhanced T1-weighted images (T1WI-CE) and fluid-attenuated inversion recovery (FLAIR) images are most widely used in radiomics [25, 26]. Because T1WI-CE requires injection of gadolinium-containing contrast agent, which may lead to nephrogenic systemic fibrosis in patients with renal insufficiency [27], the value of FLAIR image-based radiomics in the diagnosis and prognosis of GBM is currently receiving more attention. In recent years, it has been shown that prognostic models based on radiomics perform better than prognostic models with clinical factors [28, 29]. However, the correlation between radiomics and immune cell infiltration and ICB in GBM is not yet known.

The integrated study of immune-related lncRNAs, radiomics and clinical factors is expected to describe the biological processes associated with the disease more precisely and contribute to a comprehensive understanding of the interrelationship between molecular immune features of tumors and sample phenotypes. Therefore, this study constructs immune-related lncRNAs signature and radiomics signature, explores the correlation between the two signatures and immune cell infiltration and ICB in GBM. Integrates the two signatures and clinical risk factors to construct a multifactorial prognostic model and performs perfect model validation, which can determine the immune status of GBM and provide an individualized survival probability for each patient.

## Methods

### Study population

**Fig. 1:**
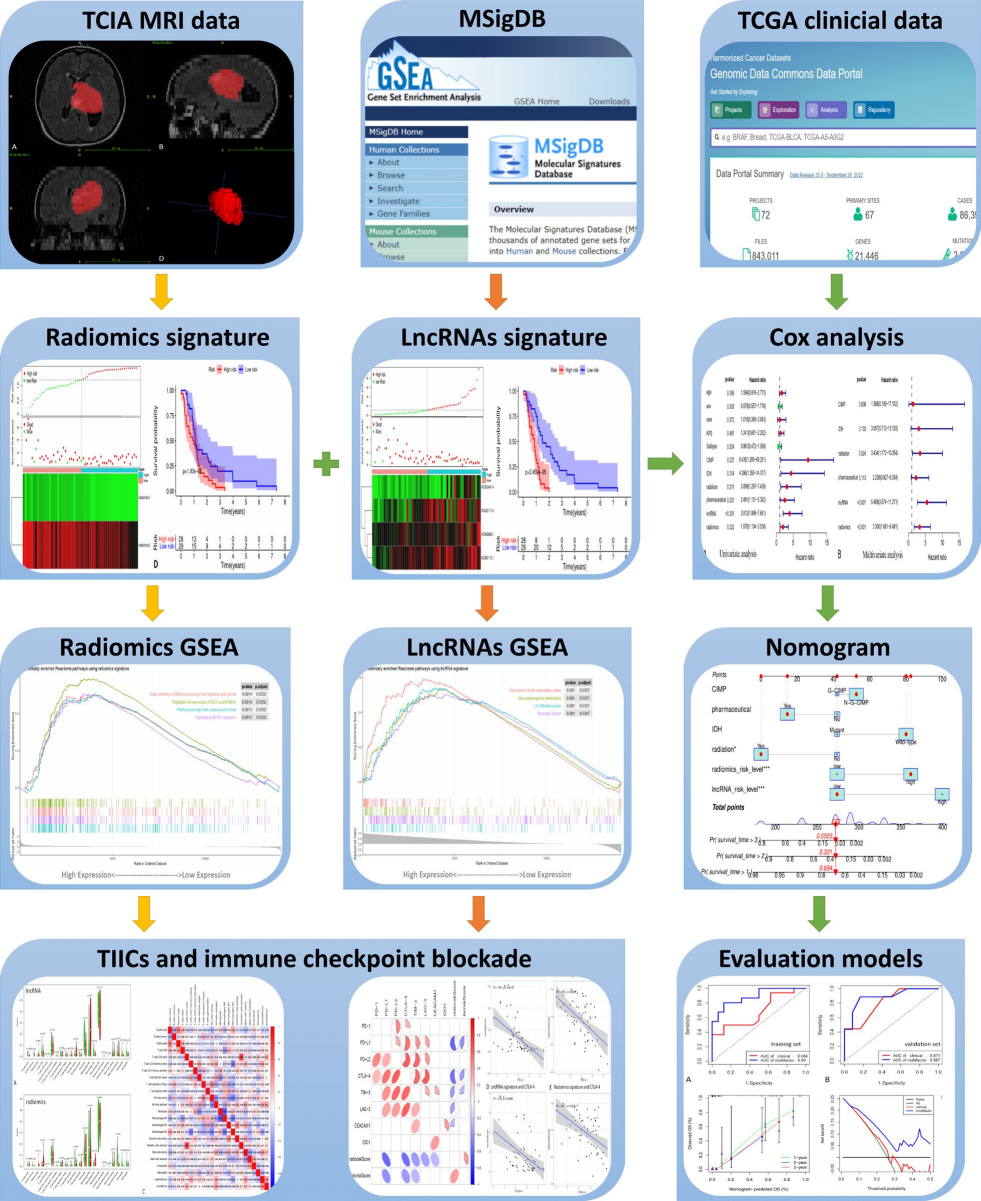
Flow chart of the analysis. The TCGA GBM RNA-seq data, TCIA MRI data and clinical information were analyzed. Immune-related lncRNA and radiomics signatures were established through Cox regression. Pathways were explored using GSEA, TIICs were estimated using CIBERSORT, and the relationship between immune checkpoint genes was investigated. A multifactors prognostic model was constructed and compared with the clinical model

Inclusion criteria for the study sample were: (1) having transcriptional information from The Cancer Genome Atlas (TCGA, https://portal.gdc.cancer.gov/), clinical information (e.g., patient gender, age, overall survival time, etc.) and (2) MRI data from The Cancer Imaging Archive (TCIA, https://www.cancerimagingarchive.net/), with a total of 174 samples enrolled. The exclusion criteria for the study samples were: (1) samples with TCGA transcriptome data and TCIA MRI data, (2) TCIA MRI data of high quality without artifacts, and (3) complete information on clinical indicators. A total of 62 samples (57 GBM patients and 5 controls) were obtained after screening and were retrospectively included in this study. The 57 patients were then randomly divided into a training set (n = 35) and a validation set (n = 22) in a ratio of 6:4. The relevant policies of TCGA and TCIA were followed in the acquisition and use of the data. In this retrospective study, the requirement for informed consent was waived as the relevant patient data in the TCGA was publicly available. The flow chart of this study is shown in Fig. 1.

### Immune-related lncRNAs acquisition

The genes related to immune system process M13664 and immune response M19817 were downloaded from the Molecular Signatures Database (http://www.broadinstitute.org/gsea/msigdb/ index.jsp) [30, 31]. The Pearson correlation coefficients of immune genes and lncRNAs were calculated. The immune-related lncRNAs were those with the absolute value of the correlation coefficient (|correlation|, |cor|) ≥ 0.4 and P < 0.01. If cor is positive it indicates a positive regulatory relationship, and if cor is negative indicates a negative regulatory relationship [32].

### Image segmentation and radiomics feature selection

ITK-SNAP (https://www.itk-snap.org/) software was used to segment the FLAIR images of patients in 3D space. The FLAIR scan parameters were as follows: thickness = 4 ~ 5.5 mm, TR/TE = 9000 ~ 12500/140 ~ 157 ms, slice gap = 4 ~ 6.5 mm, flip angle = 80 ~ 90°. All radiomics features were extracted using Pyradiomics extractor in python 3.7 (https://pyradiomics.readthedocs.io/en/latest/). To confirm the reproducibility of the features, two neuro-radiologists (reader 1: with 5 years of experience; reader 2: with 7 years of experience) performed the Region Of Interests (ROIs) segmentation on 30 samples that were randomly selected from the training set. The intraclass correlation coefficient (ICC) was calculated to evaluate the reproducibility of the values measured by the two neuro-radiologists [33]. A threshold of ICC > 0.75 was set for considering a good agreement between the two neuro-radiologists. Features that achieved ICC higher than this thereshold were considered as showing reproducibility.

### Construction of immune-related lncRNAs and radiomics signature

The univariate cox analysis was first performed for immune-related lncRNAs and radiomics features respectively, in which radiomics features with P-values less than 0.05 and immune-related lncRNAs with P-values < 0.01 were selected for multivariate Cox analysis. Factors with P < 0.05 were considered as independent prognostic associated GBM and used to construct the immune-related lncRNAs signature and radiomics signature. The risk score for the immune-related lncRNAs or radiomics signature of each patient were calculated based on the β value of the selected factors. Patients were categorized into high-risk or low-risk groups based on the median risk score. Immune-related lncRNAs and radiomics survival curves were developed to show the survival status of high-risk and low-risk patients. A multifactorial nomogram was constructed combining immune-related lncRNAs signature, radiomics signature and clinical parameters to allow clinicians to easily and accurately predict the survival of GBM patients.

### Gene set enrichment analysis

Gene set enrichment analysis (GSEA) was used to analyze significant functional phenotypes in the high-risk group and low-risk group labeled with immune-related lncRNAs signature and the radiomics signature. Reactome pathway enrichment analysis was performed using the R package clusterProfiler. Gene set permutations were performed 1000 times for each analysis to obtain a normalized enrichment score (NES), which was used for sorting pathways enriched in each phenotype. Gene sets with adjusted p-value < 0.05 were considered as significant.

### Immune cell infiltration

We used the CIBERSORT method to investigate the fraction of the 22 immune cell types in each derived phenotype and identify the characteristics of infiltrating cells in the GBM microenvironment [34]. These 22 immune cell types mainly include myeloid subtypes, NK cells, plasma cells, naive and memory B cells and T cells. Violin plots and correlation heat maps were generated to show differences in the infiltration of immunocytes between the high- and low-risk groups and to explore whether the immune-related lncRNAs and radiomics signature may play a crucial role in immune infiltration in GBM.

### Role of risk score in immune checkpoint blockade treatment

The difference of immune checkpoint inhibitor treatment in malignant tumor is related to the difference of immune checkpoint gene expression [35]. We investigated eight genes previously reported as key targets of immune checkpoint inhibitors: PD-1, PD-L1, programmed death ligand 2 (PD-L2), cytotoxic T-lymphocyte antigen 4 (CTLA-4), T-cell immunoglobulin domain and mucin domain-containing molecule-3 (TIM-3), lymphocyte activation gene 3 (LAG-3), carcinoembryonic antigen-related cell adhesion molecule 1 (CEACAM1) and indoleamine 2,3-dioxygenase 1 (IDO1) [36, 37]. To explore whether both signatures could predict the response of ICB therapy, we analyzed the correlation of immune checkpoint blockade-related key genes with immune-related lncRNAs signature and radiomics signature.

### Development and evaluation of different prognostic models

Two different prognostic models were constructed, a clinical prognostic model based on clinical candidate prognostic risk factors, including age, gender, race, Karnofsky performance score (KPS), and isocitrate dehydrogenase (IDH) typing, and a multifactorial prognostic model based on clinical candidate prognostic risk factors, immune-related lncRNAs signature, and radiomics signature. The constructed clinical prognostic models were internally validated by iterative extraction in the training set using tenfold cross-validation. External validation was then performed with the validation dataset. The predictive performance of the prognostic models was evaluated in terms of discrimination, calibration and clinical effectiveness according to Transparent Reporting of a Multivariable Prediction Model for Individual Prognosis or Diagnosis (TRIPOD) [38]. Area under the receiver-operating-characteristics (ROC) curve (AUC), the concordance index (C-index), the integrated discrimination improvement (IDI) [39] and the net reclassification improvement (NRI) [40] to evaluate the discrimination of the model. The calibration of the model is evaluated by calibration curves. The clinical utility of the prognostic models was determined by the decision curve analysis (DCA) after calculating the net benefits for patients at different risk threshold probabilities [41].

### Statistical analysis

Statistical analyses were all performed using R 3.6.0 (http://www.R-project.org, 2019). The R packages used were as follows: the limma package for calculating Pearson correlation coefficients of immune-related lncRNAs, survival package for survival analysis, ROC results obtained from the timeROC package. Survival curves were plotted using the Kaplan–Meier method and compared by log-rank test. The comparison of patients between training and validation set was performed for continuous variables with a t-test or Mann–Whitney test, and the chi-square test was performed for subtype variables, and the Fisher’s exact test was added if there were cells with theoretical frequencies less than 5. All statistics were two-tailed, and p-values less than 0.05 were considered statistically significant.

## Results

### Clinical characteristics of the patients

The clinical characteristics of the patients in the training and validation sets are shown in Table 1. There were no statistically significant differences in patient age, gender, race, whether they received radiotherapy, medication, or overall survival between the the training and validation set (P = 0.187–1.000).

**Table 1:** Demographics of the patients enrolled in the training and validation sets

Characteristics	Training set (35)	Validation set (22)	P-value
Age (years)			0.187
≤ 60	16 (45.71%)	14 (63.64%)	
> 60	19 (54.29%)	8 (36.36%)	
Gender			0.339
Female	17 (48.57%)	7 (31.82%)	
Male	18 (51.43%)	15 (68.18%)	
Race			0.946
Others	3 (8.57%)	2 (9.09%)	
White	32 (91.43%)	20 (90.91%)	
KPS score			0.538
> 60	25 (71.43%)	14 (63.64%)	
≤ 60	10 (28.57%)	8 (36.36%)	
Subtype			0.915
Classical	10 (28.57%)	6 (27.27%)	
N-Classical	25 (71.43%)	16 (72.73%)	
CIMP			0.635
G-CIMP	2 (5.71%)	2 (9.09%)	
NON G-CIMP	33 (94.29%)	20 (90.91%)	
IDH			1.000
Mutant	4 (11.43%)	2 (9.09%)	
Wild-type	31 (88.57%)	20 (90.91%)	
Radiotherapy			1.000
Yes	4 (11.43%)	2 (9.09%)	
No	31 (88.57%)	20 (90.91%)	
Pharmaceutical			1.000
Yes	5 (14.29%)	3 (13.64%)	
No	30 (85.71%)	19 (86.36%)	
Status			1.000
Alive	3 (8.57%)	2 (9.09%)	
Dead	32 (91.43%)	20 (90.91%)	
Survival time	1.24 ± 1.23	1.62 ± 1.55	0.261

### Immune-related lncRNAs and and radiomics features

A total of 331 immune-related genes were extracted from the Molecular Signatures Database v4.0, which were associated with immune response and immune system process. Next, 1286 immune-related lncRNAs were extracted using Pearson correlation analysis (|cor|≥ 0.4, p < 0.01). The relationship between the top five immune-related lncRNAs and immune genes in terms of |cor| value is shown in Table 2. 851 handcrafted radiomics features were extracted, where 107 were from the original images and 744 were from the wavelet filtered images. Univariate analysis identified six radiomics features with P values less than 0.05 as possible independent prognostic factors (Table 3).

**Table 2:** Relationship between immune-related lncRNAs and immune genes

immuneGene	lncRNAs	Cor	P value	Regulation
ARHGDIB	PCED1B-AS1	0.910	6.15E−66	Postive
TGFB2	TGFB2-AS1	0.917	1.42E−68	Postive
CTSS	AC109826.1	0.917	1.33E−68	Postive
CD24	AL355297.4	0.926	2.47E−72	Postive
CD24	LINC02526	0.986	1.33E−13	Postive
ANXA11	LINC00665	− 0.674	9.38E−24	Negative
PRELID1	LINC02035	− 0.620	2.50E−19	Negative
RPS19	BAIAP2-DT	− 0.619	2.96E−19	Negative
SART1	CARD8-AS1	− 0.606	2.64E−18	Negative
PRELID1	AP003486.1	− 0.600	6.41E−18	Negative

**Table 3:** Radiomics features associated with poor prognosis

Radiomics feature	P-value
log-sigma-3–0 mm-3D_gldm_LargeDependenceLowgrayLevelEmphasis	0.040
log-sigma-3–0 mm-3D_glrlm_LongRunLowGrayLevelEmphasis	0.044
log-sigma-3–0 mm-3D_glszm_LargeAreaHighGraLlevelEmphasis	0.034
wavelet-HLH_glszm_LargeAreaHighGrayLevelEmphasis	0.025
wavelet-LHH_glszm_LargeAreaHighGrayLevelEmphasis	0.036
wavelet-LLH_gldm_GrayLeveINonUniformity	0.032

### Construction of immune-related lncRNAs and radiomics signature

Univariate analysis was performed on immune-related lncRNAs, and 9 immune-related lncRNAs with P-values < 0.01 were obtained. Multivariate Cox regression analysis was performed on these features, and finally 4 immune-related lncRNAs with P-values < 0.01 were obtained, namely AC025171.5, AC068888.1, AC080112.1, AC002401.4. Univariate analysis was performed on the radiomics features, and six radiomics features with P values < 0.05 were obtained. Multivariate Cox regression analysis was performed on these features, and finally two radiomics features with P values < 0.05 were obtained, which were log-sigma-3–0-mm-3D_gldm_LargeDependenLowGrayLevelEmphasis, log-sigma-3–0-mm-3D_glszm_Large AreaHighGrayLevelEmphasis (Table 4).

**Table 4:** Immune-related lncRNAs and radiomics signature

Radiomics signature			LncRNAs signature		
Radiomics features	Coefficient	P-value	LncRNAs	Coefficient	P-value
log-sigma-3–0 mm-3D_gldm_LargeDependenceLowGrayLevelEmphasis	− 2.66E-02	0.026	AC025171.5	1.021	P < 0.01
			AC068888.1	1.443	P < 0.01
log-sigma-3–0 mm-3D_glszm_LargeAreaHighGrayLevelEmphasis	− 2.22E-09	0.021	AC080112.1	1.061	P < 0.01
			AC002401.4	1.191	P < 0.01

### Risk and survival curves for immune-related lncRNAs and radiomics signature

**Fig. 2:**
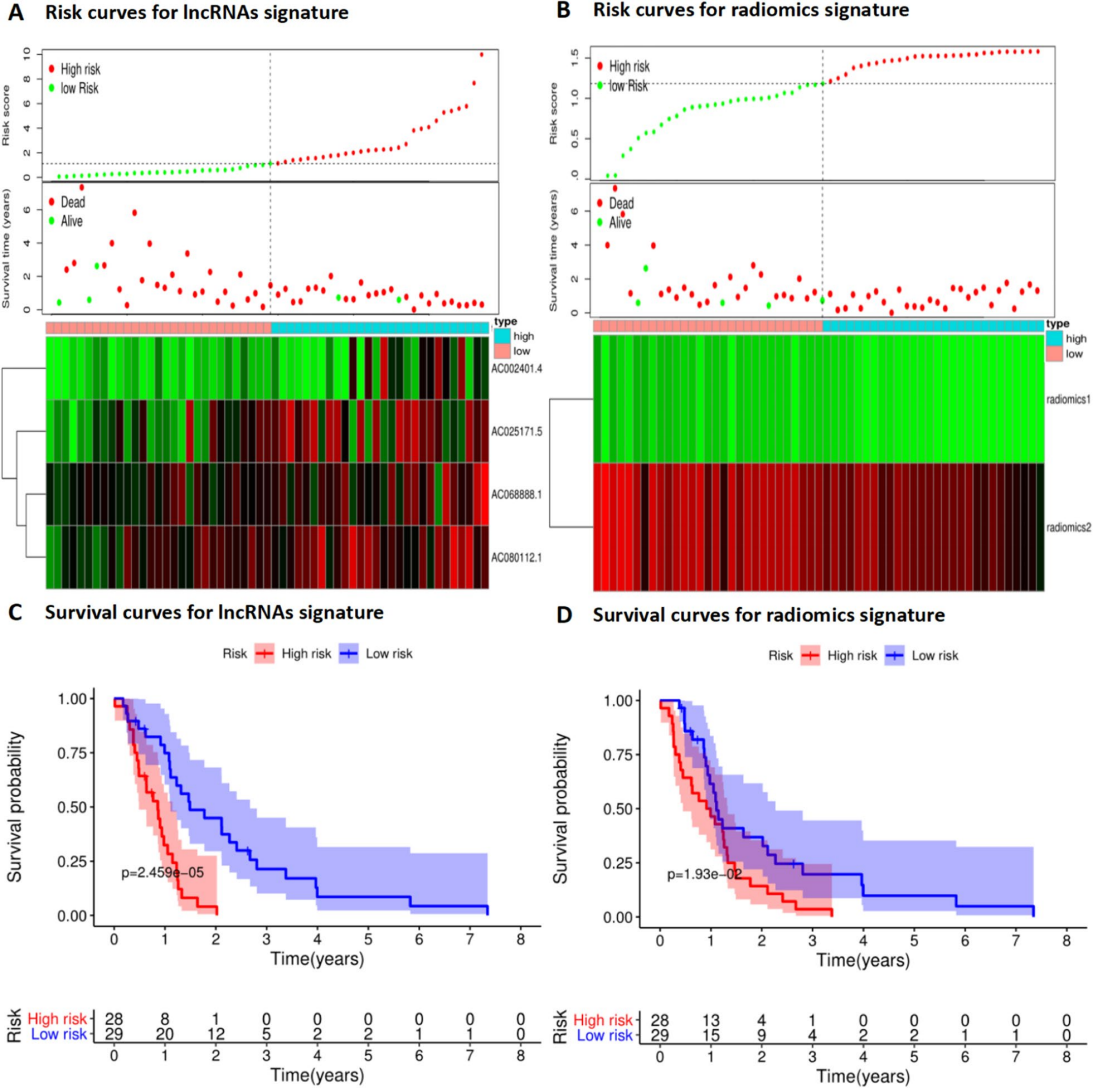
Risk curves and survival curves for immune-related lncRNAs signature and radiomics signature. **A** The distribution of risk scores, survival status and immune-related lncRNAs expression for immune-related lncRNAs in GBM patients. **B** The distribution of risk scores, survival status and radiomics values for radiomics features in GBM patients. **C** Kaplan–Meier survival curves for high and low-risk groups based on median immune-related lncRNAs risk score (log-rank test P < 0.05). **D** Kaplan–Meier survival curves for high and low-risk groups based on median radiomics risk score (log-rank test P < 0.05)

Risk scores for immune-related lncRNAs signature and radiomics signature were calculated, and patients were classified into high-risk or low-risk groups based on the median risk score. The survival time of patients decreases progressively as the risk score increases (Fig. 2A, B). Kaplan–Meier curves were applied to show the survival status of high-risk and low-risk patients, demonstrating differences in overall survival. (Fig. 2C, D), with Log-rank test P values < 0.05.

### Gene enrichment for immune-related lncRNAs and radiomics signature

**Fig. 3:**
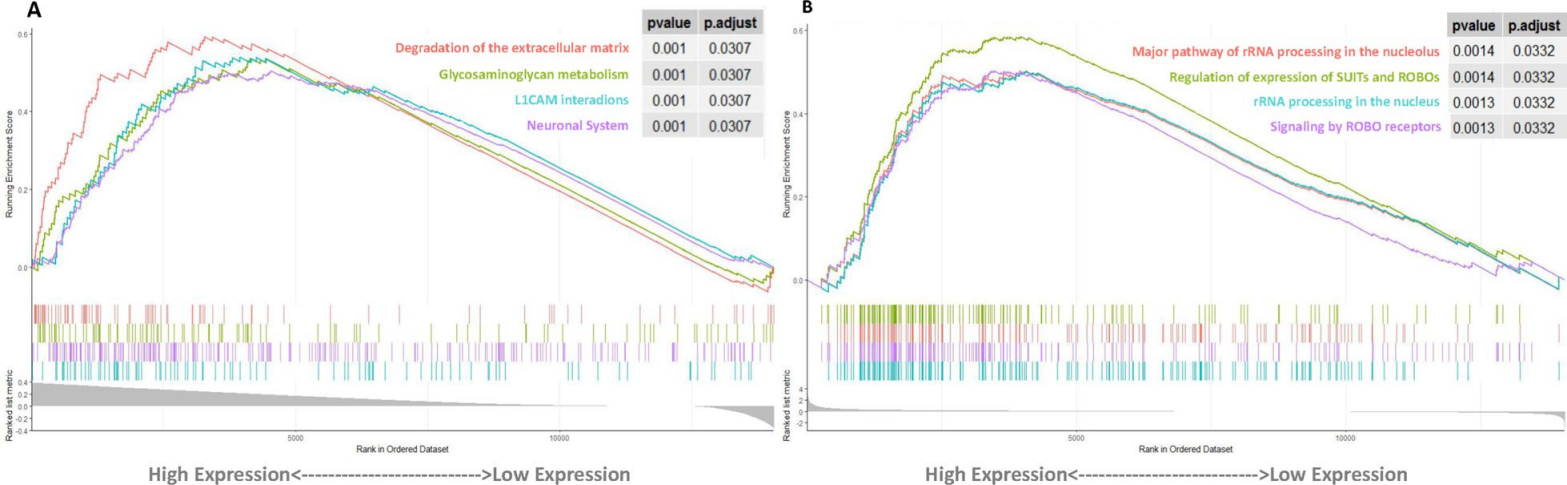
Differentially enriched Reactome pathways. **A** Differentially enriched Reactome pathways using lncRNAs signature (P < 0.05). **B** Differentially enriched Reactome pathways using radiomics signature (P < 0.05)

To explore the underlying mechanism of immune-related lncRNAs and radiomics signatures associated with GBM progression, we conducted the GSEA of the differential expression of high- and low-risk score groups. Reactome pathway enrichment analysis indicated that neuronal pathways (L1 cell adhesion molecule (L1CAM) interactions, Neuronal System) were significantly enriched in the high-risk immune-related lncRNAs group (Fig. 3A). Reactome pathway enrichment analysis indicated that neuronal pathways (Regulation of expression of SLITs and ROBOs, Signaling by ROBO receptors) were significantly enriched in the high-risk radiomics group (Fig. 3B).

### The relationship between signatures and immune cell infiltration

**Fig. 4:**
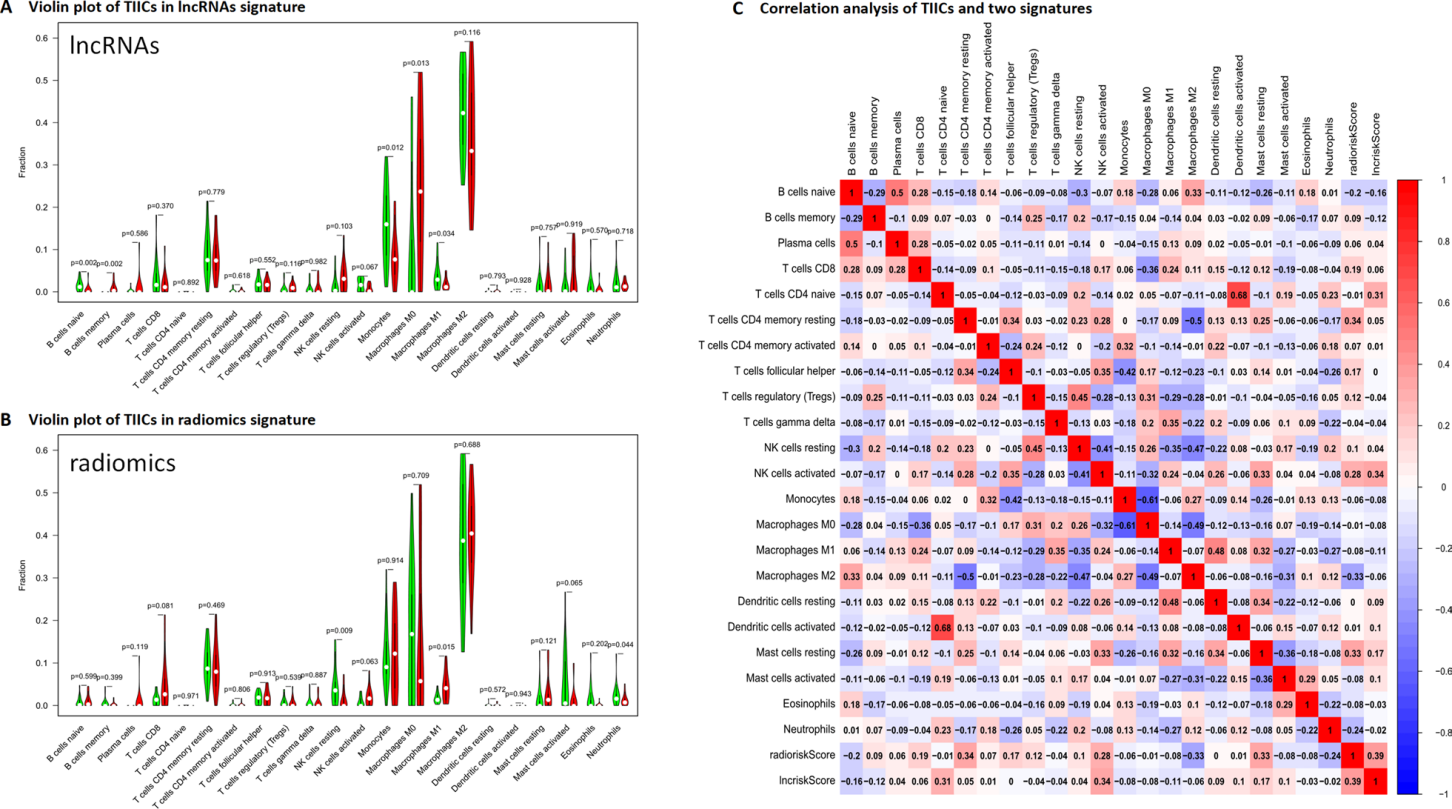
Immune-related lncRNAs signature, radiomics signature and relationship with immune cell infiltration. **A** Violin plot showed high- and low-risk groups identified by immune-related lncRNAs signature, red for high risk and green for low risk; **B** Violin plot showed high- and low-risk groups identified by radiomics signature, red for high risk and green for low risk; **C** Correlation heat map illustrated the the correlation between the two risk score and immune cell infiltration

Next, we assessed the relationship between the immune-related lncRNAs signature and radiomics signature and tumor immune microenvironment in GBM. Immune cell infiltration was obtained using CIBERSORT and the correlation between the two risk score and immune cell infiltration was analyzed. In the immune-related lncRNAs signature, we observed higher expression of B cells memory and Macrophages M0 in the high-risk group compared to the low-risk group (P < 0.05), while B cells naive, Monocytes, and Macrophages M1 showed lower levels in the high-risk group than in the low-risk group (P < 0.05) (Fig. 4A). In the radiomics signature, we observed higher expression of Macrophages M1 in the high-risk group compared to the low-risk group (P < 0.05), while NK cells resting and Noutrophils showed lower levels in the high-risk group than in the low-risk group (P < 0.05) (Fig. 4B). We next analyzed the correlation of the risk score to tumor microenvironment and the two risk score in GBM. We found positive correlation with radiomics score and T cells CD4 memory resting (cor = 0.34, P < 0.05), and positive correlation with immune-related lncRNAs score and NK cells activated (cor = 0.34, P < 0.05). Meanwhile, we found positive correlation with radiomics score and immune-related lncRNAs score (cor = 0.39, P < 0.05) (Fig. 4C).

### The relationship between signatures and immune checkpoint blockade

**Fig. 5:**
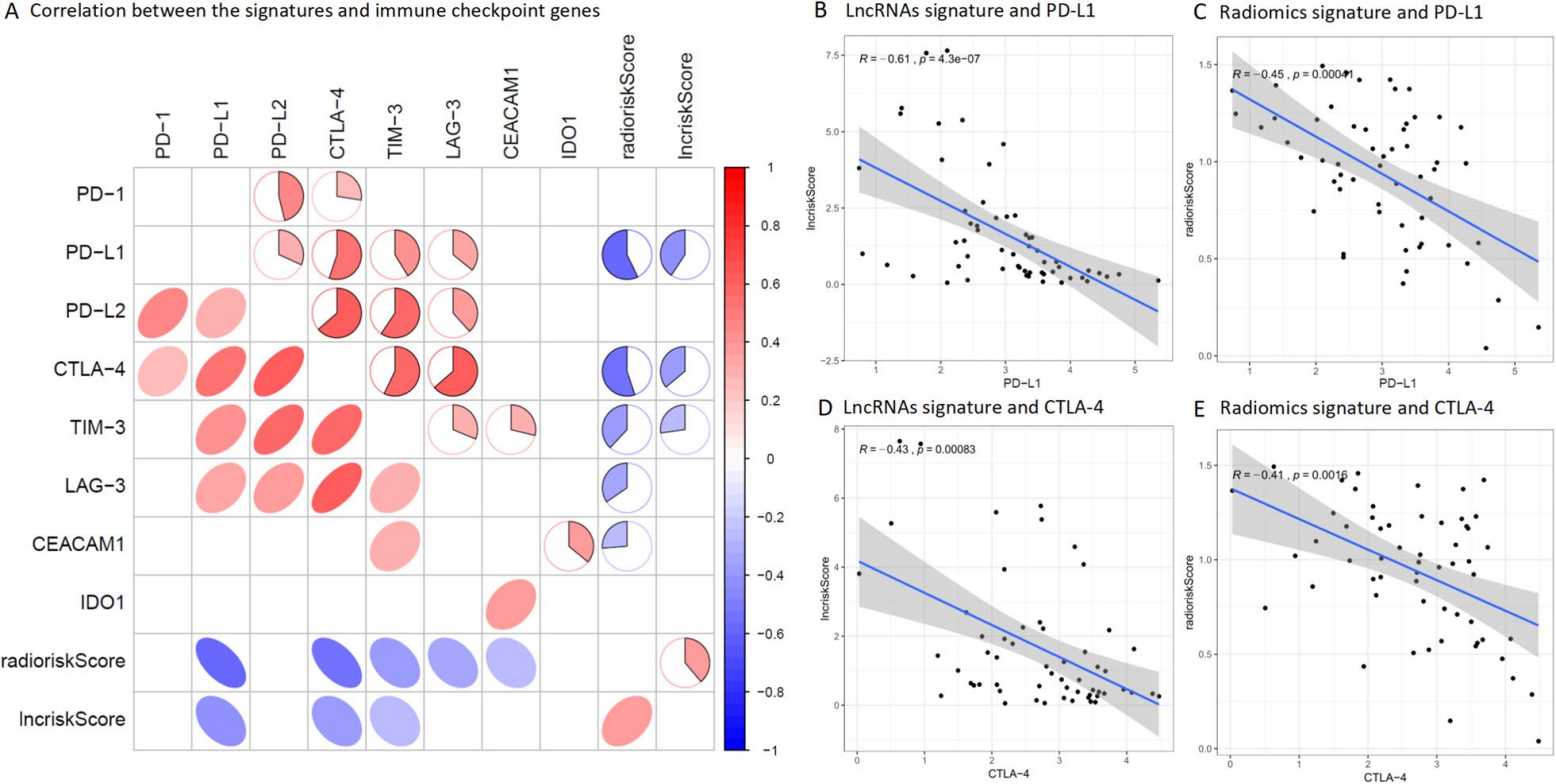
Immune-related lncRNAs signature, radiomics signature and relationship with immune checkpoint genes. **A** Associations between the two signatures and immune checkpoint genes were detected, red circles indicate positive correlation and blue circles indicate negative correlation. **B–E** Associations between the two signatures and PD-L1, CTLA-4 were detected (P < 0.05)

The application of ICB for immunotherapy has become a promising aid to the treatment of various cancers. Therefore, we investigated the possible role of our Immune-related lncRNAs signature, radiomics signature in the ICB therapy of GBM by evaluating the relationships of the eight well known targets of immune checkpoint inhibitors (including PD-1, PD-L1, PD-L2, CTLA-4, TIM-3, LAG-3, CEACAM1, IDO1) to the immune-related lncRNAs signature and radiomics signature (Fig. 5A). We found negative correlation with immune-related lncRNAs score and PD-L1 (cor = − 0.61; P < 0.05), and negative correlation with the score and CTLA-4 (cor = − 0.43; P < 0.05), and negative correlation with radiomics score and PD-L1 (cor = − 0.45; P < 0.05), and negative correlation with the score and CTLA-4 (cor = −  0.41; P < 0.05) (Fig. 5B–E).

### The prognostic value of immune-related lncRNAs and radiomics signature

**Fig. 6:**
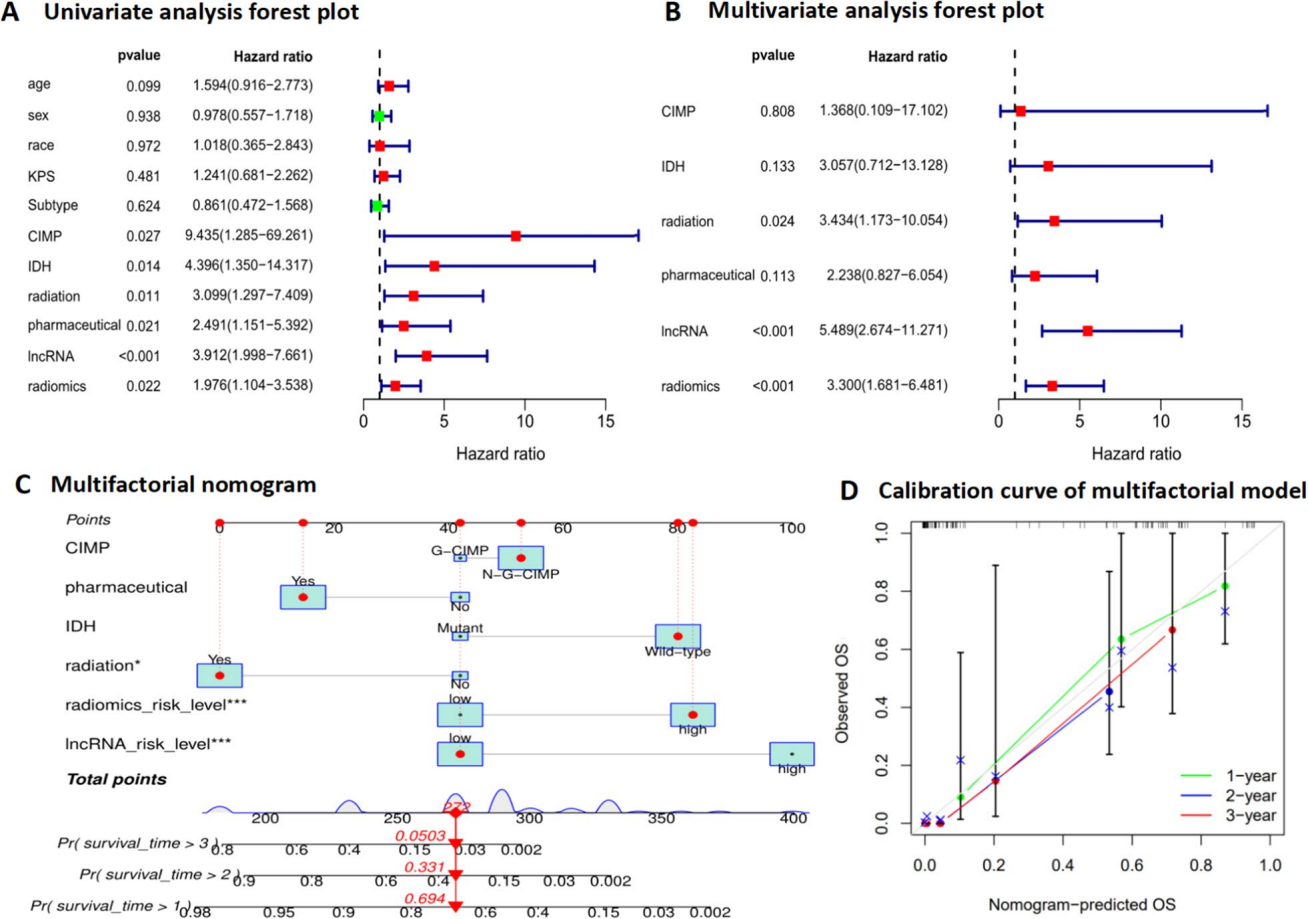
Forest plot of Cox regression analysis and the multifactorial nomogram. **A** Forest plot of univariate Cox regression analysis, CIMP, radiation, pharmaceutical, lncRNAs, radiomics had a statistically significant effect on prognosis, P < 0.05. **B** Forest plot of multivariate Cox regression analysis, radiation, lncRNAs and radiomics had a statistically significant effect on prognosis, P < 0.05. **C** The multifactorial nomogram for predicting survival in GBM patients. **D** The calibration plots of multifactorial nomogram for predicting survival in GBM patients

Univariate Cox analysis showed that CIMP (HR = 9.435; P = 0.027), IDH (HR = 4.396; P = 0.014), radiation (HR = 3.099; P = 0.011), pharmaceutical (HR = 2.491; P = 0.021), immune-related lncRNAs risk level (HR = 3.912; P < 0.01) and radiomics risk level (HR = 1.976; P = 0.022) were prognostic factors for overall survival in GBM (Table 5); multivariate Cox analysis showed that radiation (HR = 3.434; P = 0.024), immune-related lncRNAs risk level (HR = 5.489; P < 0.01) and radiomics risk level (HR = 3.300; P < 0.01) were prognostic factors for overall survival in GBM (Table 5). The forest plot of the cox regression is shown in Fig. 6A, B. Prognostic factors with P < 0.05 in univariate Cox analysis were included in the multifactorial nomogram, combining lncRNAs signature, radiomics signature and clinical factors of the multifactorial nomogram is shown in Fig. 6C. Using the nomogram, the 1-, 2- and 3 year survival probabilities of GBM patients can be easily predicted by adding the points of the predictors. And the calibration plots of this model showed better calibration performance (Fig. 6D).

**Table 5:** Univariate and multivariate Cox analysis of overall survival in GBM patients

Variables	Univariate analysis		Multivariate analysis	
	Hazard ratio (95% CI)	p value	Hazard ratio (95% CI)	p value
Age (> 60)	1.594 (0.916–2.773)	0.099		
sex (Male)	0.978 (0.557–1.718)	0.938		
race (White)	1.018 (0.365–2.843)	0.972		
KPS (≤ 60)	1.241 (0.681–2.262)	0.481		
Subtype (Non-classical)	0.861 (0.472–1.568)	0.624		
CIMP (Non G-CIMP)	9.435 (1.285–69.261)	0.027	1.368 (0.109–17.102)	0.808
IDH (Wild-type)	4.396 (1.350–14.317)	0.014	3.057 (0.712–13.128)	0.133
radiation (No)	3.099 (1.297–7.409)	0.011	3.434 (1.173–10.054)	0.024
pharmaceutical (No)	2.491 (0.185–5.392)	0.021	2.238 (0.827–6.054)	0.113
lncRNAs_risk_level (High)	3.912 (1.998–7.661)	< 0.001	5.489 (2.674–11.271)	< 0.001
radiomics_risk_level (High)	1.976 (1.104–3.538)	0.022	3.300 (1.681–6.481)	< 0.001

### Evaluation of the prognostic models

**Fig. 7:**
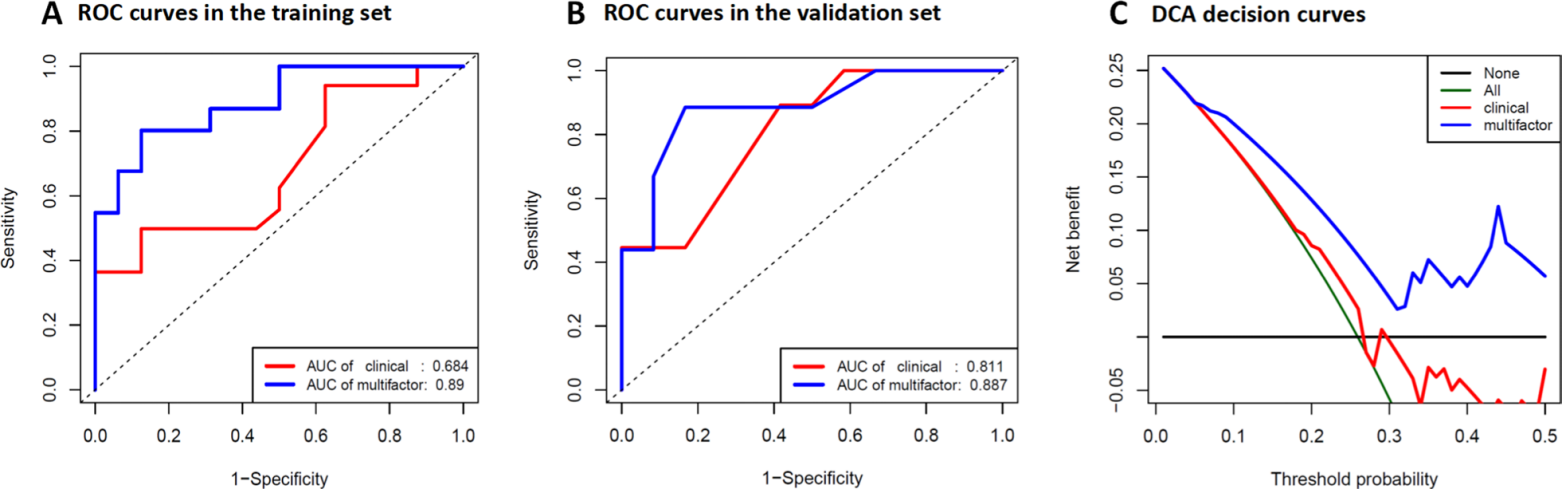
ROC curves and DCA decision curves for different prognostic models. **A**, **B** The ROC curves for the multifactorial prognostic model and the clinical prognostic model in the training set and validation set; the blue line represents the multifactorial prognostic model and the red line represents the clinical prognostic model. **C**. DCA decision curves, the blue line represents the multifactorial prognostic model and the red line represents the clinical prognostic model

Compared to the clinical prognostic model, the AUC values (training set: 0.890 vs. 0.684, validation set: 0.887 vs. 0.811) and the C-index (training set: 0.737 vs. 0.658, validation set: 0.817 vs. 0.807) of the multifactorial prognostic model exhibited better predictive performance (Table 6). IDI and NRI are indicators used in statistics to evaluate the performance improvement of predictive models, and a positive result indicates that the new model has improved compared to the old model. When immune-related lncRNAs and radiomics signatures were added to the clinical prognostic model, the IDI was 0.071 and the NRI was 1.327 in training set, the IDI was 0.063 and the NRI was 0.693 in validation set, indicating strong reclassification improvement (Table 6). Additionally, the DCA curves yielded larger net benefits than the traditional clinical prognostic model (Fig. 7C). The curve showed that the multifactorial prognostic model had a higher overall net benefit than the clinical prognostic model, within the threshold probability < 0.5.

**Table 6:** Discriminative index of different prognostic models in training and validation sets

Indicators	Classification	Training set	Validation set
AUC	Clinical prognostic model	0.684	0.811
	Multifactorial prognostic model	0.890	0.887
C-index	Clinical prognostic model	0.658	0.807
	Multifactorial prognostic model	0.737	0.817
IDI	Multifactorial vs. Clinical prognostic model	0.071	0.063
NRI	Multifactorial vs. Clinical prognostic model	1.327	0.693

## Discussion

GBM is a common, heterogeneous and very aggressive malignant primary brain tumor. The immune status in the tumor microenvironment is involved in cancer progression, metastasis and recurrence and is an important cause of poor tumor prognosis. Studies have shown that immune-related lncRNAs and radiomics features can be used as biomarkers of GBM and provide potential information for the treatment and prognosis of GBM. However, whether immune-related lncRNAs and radiomics features correlate with the immune status in the tumor microenvironment is not yet known. Therefore, in this study, based on TCGA and TCIA, an immune-related lncRNAs signature consisting of four lncRNAs and an radiomics signature consisting of two radiomics features were constructed to explore the correlation between the two signatures and GBM immune cell infiltration and ICB. A multifactorial prognostic model was developed and validated, which can predict the overall survival of GBM patients and guide the selection of immunotherapy for patients.

A large variety of lncRNAs are expressed in brain tissue and are emerging as key regulators of neuronal function and diseases [42]. LncRNAs have been shown to be potential targets for cancer therapy and have predictive value for survival prognosis [30, 43–46]. The immune-related lncRNAs signature constructed in this study consisted of four lncRNAs, and univariate Cox analysis showed that it was strongly associated with GBM survival. Meanwhile, the immune-related pathways in the immune-related lncRNAs high-risk groups included L1CAM interaction, neurological and other pathways. L1CAM was found to be a significant marker in predicting the prognosis of glioma patients, and may be a promising therapeutic target and monitoring index in glioma patients [47]. The immune-related lncRNAs signature in this study was positively correlated with NK cells activated. Blockade of interleukin 2 (IL-2) triggering of tumor-derived NK-cells are necessary to enhance NK-cell responsiveness in GBM [48]. As of now, the most widely recognized checkpoint molecules include PD-1, PD-L1, and CTLA-4 [18]. The immune-related lncRNAs signature of this study was associated with ICB immunotherapy key genes (PD-L1 and CTLA-4). It was found that the incidence of PD-L1 expression in GBM patients is frequent, and higher expression of PD-L1 is correlated with worse outcome [49]. Monitoring of regulatory T cell frequencies and expression of CTLA-4 on T cells, can predict survival in GBM patients [50]. This suggests that the signature may have a role in predicting the response of GBM to immunotherapy. LncRNAs and miRNAs both belong to non-coding RNA and can become potential biomarkers for cancer diagnosis and prognosis evaluation [5]. Some scholars have used lncRNAs to construct prognostic signature to assess GBM prognosis. For example, Zhang et al. [16] identified a 6-lncRNAs signature with prognostic value in GBM by mining lncRNAs expression profiling in 213 GBM tumors from TCGA. Gao et al. [51] suggests that the lncRNAs signature could serve as novel biomarkers for predicting prognosis and treatment outcome of postoperative GBM patients. These findings showed that the expression of lncRNAs can be used as a molecular biomarker for prognosis or ICB therapy stratification of GBM.

Radiomics analysis, which converts medical images into mineable high-dimensional data, is a promising method for the noninvasive assessment of tumors [17]. MRI plays an important role in the prognostic assessment of GBM, with enhancement scans and FLAIR being the most widely used [26]. Some of the progressive patients showed no significant enhancement on enhancement scan, but showed high signal on FLAIR sequence [52]. The results of the present study showed that 2 radiomics features obtained on FLAIR sequences were closely associated with GBM survival, which were derived from the GLDM and GLSZM, respectively. These features indicated gray-scale heterogeneity of GBM. Some studies have used radiomics to explore the gene phenotype of GBM, but they have not been related to immune lncRNAs and immune cell infiltration [25, 53, 54]. For example, Wang et al. [53] used diffusion tensor imaging group study to investigate the biological underpinnings of IDH wild-type glioblastoma. Our study indicates that the immune-related pathways in the radiomics high-risk groups included Regulation of expression of SLITs and ROBOs, Signaling by ROBO receptors. It has been proved that SLIT2/ROBO1 signaling inhibits glioma cell migration and invasion by inactivation of Cdc42-GTP [55]. The radiomics signature in this study was positively correlated with NK cells activated, T cells CD4 memory resting and immune-related lncRNAs signature. Study showed that tumors are highly enriched in M2 macrophages, resting memory CD4 + T cells, and activated dendritic cells, indicating that they may be ideal candidates for immunotherapy [56]. Our study demonstrates that radiomics signature can assess the immune cell infiltration status of GBM. Previous studies have shown that radiomics features could capture the hidden relationships between immune cell infiltration signatures and imaging phenotypes [19]. The present study showed that eight ICB treatment key target genes were co-expressed, and among these co-expressed pairs, most of them were significantly and positively correlated, which is similar to the previously reported results of co-expression of key target genes of immune checkpoint inhibitors in melanoma [57]. Furthermore, our risk signatures were significantly associated with the ICB treatment key target genes. However, our study also presented some genes that did not correlate with our risk signatures, probably due to the small number of our samples.

In terms of discrimination, calibration, and clinical validity, this multifactor prognostic model outperformed the clinical factor model. Only clinical factors have been included in most current studies for the construction of prognostic models [58–60]. Some researchers have developed prognostic models using lncRNAs. For example, Zhou et al. [61] developed an immune-related prognostic model using lncRNAs that can divide patients into high-risk and low-risk groups with a survival analysis log-rank test P < 0.05. Additionally, some researchers have incorporated imaging features to create prognostic models. For prognostic models to evaluate the prognosis of gliomas, some researchers have also combined transcriptomic, imaging, and clinicopathological parameters. For example, Chaddad et al. [62] composited model combining radiomics features, clinical features (treatment type, age), genomics, and protein expression had the largest AUC. However, the models were not evaluated for their differentiation, calibration and clinical validity. The AUC for predicting survival was greater in both the training and validation sets compared to the clinical prognostic model in this study, which included immune-related lncRNAs and radiomics characteristics to build a multifactorial prognostic model with superior predictive ability. Additionally, DCA, NRI, and IDI analyses were applied in the current study to assess the clinical improvement of multifactorial prognostic model-assisted decisions on patient outcomes. The DCA showed that using the multifactorial prognostic model to predict OS obtains more benefits. The NRI and IDI analyses confirmed the reclassification improvement by adding lncRNAs and radiomics ignature to the clinical prognostic model.

## Limitations

The present study has some limitations that can be addressed in future work. First, because MRI data was gathered retroactively from the TCIA database, it was impossible to regulate the heterogeneity of various imaging parameters produced by various equipment and field strengths. Additionally, the study only included a small number of patients. Incomplete clinical risk factors in some cases included isocitrate dehydrogenase (IDH) mutations and O6-methylguanine DNA methyltransferase (MGMT) status. In order to produce a more precise survival prediction for GBM patients, we will perform a prospective study in the future to enroll more patients and guarantee the consistency of the scanning data. Second, due to the large amount of redundant information in the sequence images, this leads to a huge workload and subjectivity in manual segmentation. A more mature approach is to use deep learning models such as Convolutional Neural Networks (CNN) to learn features directly from images, which saves workload and reduces the presence of subjectivity. Finally, this study only extracted features on FLAIR sequences to construct a multivariate Cox prognostic model, and did not include structural images and other functional MRI techniques such as Dynamic Susceptibility Contrast Enhancement Imaging (DSC), Diffusion Tensor Imaging (DTI) into the model. In the future, we will add new imaging techniques and combine them with tumor immunology, radiomics, radiogenomics, and transcriptomics for combined analysis, in order to more accurately and objectively assess the prognosis of GBM patients.

## Conclusions

In conclusion, based on the TCGA and TCIA databases, this study identified immune-related lncRNAs signature consisting of four lncRNAs and radiomics signature consisting of two imaging features, both of which were associated with the progression and prognosis of GBM, as well as with immune cell infiltration and potential ICB immunotherapy-related genes, developing and validating the multifactorial prognostic model based two signatures and clinical information, which showed excellent performance in terms of differentiation, calibration and clinical validity. Therefore, this study provides a possible approach for individualized prognostic assessment of GBM and detection of ICB immunotherapeutic response, which has important clinical application in tumor immunotherapy.

## Data Availability

The original contributions presented in the study are included in the article. Further inquiries can be directed to the corresponding author.

## Abbreviations

GBM: Glioblastoma multiforme
LncRNAs: Long non-coding RNAs
TCGA: The Cancer Genome Atlas
TCIA: The cancer imaging archive
GSEA: Gene set enrichment analysis
TIICs: Tumor-infiltrating immune cells
L1CAM: L1 cell adhesion molecular
AUC: Area under the curve
ICB: Immune-checkpoint blockade
PD-1: Programmed death 1
PD-L1: Programmed cell death 1 ligand 1
ICIs: Immune checkpoint inhibitors
T1WI-CE: Contrast-enhanced T1-weighted images
FLAIR: Fluid-attenuated inversion recovery
MRI: Magnetic resonance imaging
ROIs: Region of interests
ICC: Intraclass correlation coefficient
NES: Normalized enrichment score
PD-L2: Programmed death ligand 2
CTLA-4: Cytotoxic T-lymphocyte antigen 4
TIM-3: T-cell immunoglobulin domain and mucin domain-containing molecule-3
LAG-3: Lymphocyte activation gene 3
CEACAM1: Carcinoembryonic antigen-related cell adhesion molecule 1
IDO1: Indoleamine 2,3-dioxygenase 1
KPS: Karnofsky performance score
IDH: Isocitrate dehydrogenase
TRIPOD: Transparent reporting of a multivariable prediction model for individual prognosis or diagnosis
ROC: Receiver operating characteristics
IDI: Integrated discrimination improvement
NRI: Net Reclassification index
DCA: Decision curve analysis
CIMP: CpG Island methylator phenotype
HR: Hazard ratio
CI: Confidence interval
EGFR: Epidermal growth factor receptor
CNN: Convolutional Neural Networks
MGMT: O6-Methylguanine DNA methyltransferase
DSC: Dynamic susceptibility contrast
DKI: Diffusion Kurtosis Imaging
